# Supplementary material for: Analysis of PPARγ Signaling Activity in Psoriasis
Source: Int J Mol Sci. 2021 Aug 10;22(16):8603. doi: 10.3390/ijms22168603 (PMC8395241; doi:10.3390/ijms22168603)
Supplement: Supplementary file 1 [file ijms-22-08603-s001.zip › Supplemental materials_Analysis of PPARg signaling activity in psoriasis/Pathway models/Models images and html files/Interleukin-17 and interleukin-22 signaling in psoriasis/142071.html]

apoptosis


# Cell Process apoptosis

|  |  |
| --- | --- |
| URN | urn:agi-gocellproc:0006915 |
| Total Entities | 167 |
| Connectivity | 27040 |
| Name | apoptosis |
| ObjectType | Cell Process |

---

|  |  |
| --- | --- |
| ChildConcepts | erythrocyte apoptosis |
|  | ferroptosis |
|  | lipoapoptosis |
|  | extrinsic pathway of apoptosis |
|  | intrinsic pathway of apoptosis |
|  | ovarian follicle atresia |
|  | neutrophil apoptosis |
|  | B cell apoptosis |
|  | mature B cell apoptosis |
|  | disassembly of cell structures |
|  | cleavage of lamin |
|  | cleavage of cytoskeletal proteins |
|  | lymphocyte apoptosis |
|  | activation-induced cell death of T-cells |
|  | inflammatory cell apoptosis |
|  | virus-infected cell apoptosis |
|  | transformed cell apoptosis |
|  | DNA fragmentation during apoptosis |
|  | viral inhibition of apoptosis |
|  | mast cell apoptosis |
|  | myeloid cell apoptosis |
|  | anoikis |
|  | nurse cell apoptosis |
|  | retinal cell apoptosis |
|  | autophagic cell death |
|  | neuron apoptosis |
|  | muscle cell apoptosis |
|  | glial cell apoptosis |
|  | smooth muscle cell apoptosis |
|  | fibroblast apoptosis |
|  | leukocyte apoptosis |
|  | thymocyte apoptosis |
|  | natural killer cell apoptosis |
|  | bone marrow apoptosis |
|  | macrophage apoptosis |
|  | endothelial cell apoptosis |
|  | dendritic cell apoptosis |
|  | motor neuron apoptosis |
|  | pancreatic beta cell apoptosis |
|  | mesenchymal cell apoptosis |
|  | oligodendrocyte apoptosis |
|  | hepatocyte apoptosis |
|  | cleavage of lamina in apoptosis |
|  | stem cell apoptosis |
|  | umbilical vein endothelial cell apoptosis |
|  | myofibroblast apoptosis |
|  | T-cell apoptosis |
|  | eosinophil apoptosis |
|  | granulocyte apoptosis |
|  | kidney mesangial cell apoptosis |
|  | immunocompetent cell apoptosis |
|  | double positive thymocyte apoptosis |
|  | capillary endothelial cell apoptosis |
|  | lung fibroblast apoptosis |
|  | bone marrow derived mesenchymal stem cell apoptosis |
|  | microglia apoptosis |
|  | monocyte apoptosis |
|  | blood platelet apoptosis |
|  | haematopoietic stem cell apoptosis |
|  | regulatory T-cell apoptosis |
|  | myeloid progenitor cell apoptosis |
|  | B-lymphoid precursor cell apoptosis |
|  | M1 macrophage apoptosis |
|  | effector T-cell apoptosis |
|  | erythroid precursor cell apoptosis |
|  | blast cell apoptosis |
|  | PBMC apoptosis |
|  | bone marrow stromal cell apoptosis |
|  | CD8+ T-cell apoptosis |
|  | cytotoxic T-cell apoptosis |
|  | immune system cell apoptosis |
|  | hematopoietic cell apoptosis |
|  | alveolar macrophage apoptosis |
|  | CD4+ T-cell apoptosis |
|  | Tfh cell apoptosis |
|  | plasma cell apoptosis |
|  | Th1 cell apoptosis |
|  | tumor infiltrating macrophage apoptosis |
|  | foam cell apoptosis |
|  | erythroid cell apoptosis |
|  | tumor-infiltrating lymphocyte apoptosis |
|  | bone marrow macrophage apoptosis |
|  | megakaryocyte apoptosis |
|  | helper T-cell apoptosis |
|  | germinal center B-cell apoptosis |
|  | brain capillary endothelial cell apoptosis |
|  | basophil apoptosis |
|  | Th2 cell apoptosis |
|  | follicular dendritic cell apoptosis |
|  | Kuppfer cell apoptosis |
|  | lymphoblast apoptosis |
|  | myeloid derived suppressor cell apoptosis |
|  | dermal microvascular endothelial cell apoptosis |
|  | Th17 cell apoptosis |
|  | myelocyte apoptosis |
|  | sinusoidal cell apoptosis |
|  | plasmacytoid dendritic cell precursor apoptosis |
|  | antigen-presenting cell apoptosis |
|  | plasmacytoid dendritic cell apoptosis |
|  | natural killer T-cell apoptosis |
|  | erythroblast apoptosis |
|  | lymphoid progenitor cell apoptosis |
|  | T-lymphoid precursor cell apoptosis |
|  | neutrophil precursor apoptosis |
|  | megakaryocyte progenitor cell apoptosis |
|  | mononuclear cell apoptosis |
|  | cytotoxic lymphocyte apoptosis |
|  | phagocyte apoptosis |
|  | bone marrow derived mast cell apoptosis |
|  | peripheral lymphocyte apoptosis |
|  | peritoneal macrophage apoptosis |
|  | granulocyte precursor cell apoptosis |
|  | fibrocyte apoptosis |
|  | Langerhans cell apoptosis |
|  | lymphokine-activated killer cell apoptosis |
|  | conventional dendritic cell apoptosis |
|  | memory T-cell apoptosis |
|  | CD4+ memory T-cell apoptosis |
|  | spleen lymphocyte apoptosis |
|  | transitional B-cell apoptosis |
|  | inducible CD4+CD25+ regulatory T-cell apoptosis |
|  | marginal zone B-cell apoptosis |
|  | colony forming cell apoptosis |
|  | activated dendritic cell apoptosis |
|  | activated B-cell apoptosis |
|  | bone marrow stem cell apoptosis |
|  | cancer-associated fibroblast apoptosis |
|  | immature B-cell apoptosis |
|  | memory B-cell apoptosis |
|  | epithelioid cell apoptosis |
|  | promyelocyte apoptosis |
|  | alphabeta T-cell apoptosis |
|  | cancer stem cell apoptosis |
|  | null cell apoptosis |
|  | pre-thymocyte apoptosis |
|  | prolymphocyte apoptosis |
|  | bone marrow derived dendritic cell apoptosis |
|  | bone marrow derived mononuclear cell apoptosis |
|  | perivascular macrophage apoptosis |
|  | M2 macrophage apoptosis |
|  | antibody-producing cell apoptosis |
|  | myeloblast apoptosis |
|  | megakaryoblast apoptosis |
|  | cytokine-induced killer cell apoptosis |
|  | eosinophil precursor apoptosis |
|  | double negative thymocyte apoptosis |
|  | gammadelta T-cell apoptosis |
|  | T-lymphoblast apoptosis |
|  | regulatory B-cell apoptosis |
|  | B-1a B-cell apoptosis |
|  | CD4+ CD25+ regulatory T-cell apoptosis |
|  | mast cell precursor apoptosis |
|  | plasmablast apoptosis |
|  | activated CD4+ T-cell apoptosis |
|  | invariant natural killer T-cell apoptosis |
|  | adipose macrophage apoptosis |
|  | reticulocyte apoptosis |
|  | effector memory CD8+ T-cell apoptosis |
|  | decidual natural killer cell apoptosis |
|  | metamyelocyte apoptosis |
|  | CD5+ T-cell apoptosis |
|  | activated CD8+ T-cell apoptosis |
|  | Th9 cell apoptosis |
|  | histiocyte apoptosis |
|  | neutrophil band cell apoptosis |
|  | naive T-cell apoptosis |
|  | CD8+ regulatory T-cell apoptosis |

---

|  |  |
| --- | --- |
| Pathway | Alzheimer's Disease |
|  | Skin Aging |
|  | Apoptosis of Cochlear Hair Cells (Noise-Induced, Mouse Model) |
|  | Melatonin Effects on Circadian Cycle |
|  | mTOR Signaling |
|  | mTOR Signaling Hyperfunction |
|  | SIRT2 Signaling in Aging |
|  | Fatty Acids Cause Oxidative Stress and Mitochondria Decline in Aging |
|  | Insulin Signaling |
|  | Prolactin Production |
|  | Estrogens in Prolactin Production |
|  | Toll-like Receptors Act through MYD88-TIRAP Signaling |
|  | TLR4 Signaling in Leukocytes |
|  | T-Cell Maturation (Hypothesis) |
|  | T-Cell Positive Selection and Neglect Induced Death |
|  | T-Cell Central Tolerance |
|  | T-Cell Cytotoxic Mediated Cell Death |
|  | CXC Chemokine Receptor Signaling |
|  | CC Chemokine Receptor Signaling |
|  | Necrosis |
|  | AHR in Intestinal Cell Antimicrobial Barrier Maintenance |
|  | Keep out Signal: LTF Inhibits Neutrophil Recruitment and Inflammation |
|  | Basophil Activation |
|  | Eosinophil Survival by Cytokine Signaling |
|  | Eosinophil Activation |
|  | Histidine-Rich Glycoprotein (HRG) |
|  | Melatonin in Cell Survival and Antioxidant Response |
|  | Nuclear Lamina Cleavage |
|  | Apoptosis |
|  | G1/S Damage Checkpoint |
|  | Cell Cycle Overiew |
|  | Tight Junction Assembly (Occludin) |
|  | SCF/FBXO11 Complex |
|  | CUL4/CRL (SCF4) Complex |
|  | SCF/BTRC Complex |
|  | SCF/SKP2 Complex |
|  | SCF/FBXO7 Complex |
|  | Scavenger Receptor OLR1 in Inflammation Related Endothelial Dysfunction |
|  | Low-Density Lipoproteins and Chemokines in Atherosclerosis |
|  | Endothelial Cell Dysfunction in Pulmonary Hypertension |
|  | Smooth Muscle Cell Dysfunction in Pulmonary Hypertension |
|  | BMP2 and Canonical WNT Signaling in Pulmonary Artery Endothelial Cells |
|  | Reperfusion Injury in Myocardial ischemia |
|  | Oxygen Shortage in Myocardial Ischemia |
|  | Myocardial Infarction |
|  | Preconditioning Ischemia |
|  | Apoptosis of Myocyte |
|  | NO Synthesis in Early Vasospasm |
|  | Hemoglobin Reduction and Leukocyte Adhesion Initiate Vasospasm |
|  | Hair Follicle Keratinocyte Apoptosis |
|  | Harlequin Ichthyosis |
|  | Melanoma |
|  | Apoptosis Block in Melanoma Cell |
|  | MITF as a Regulator of Melanoma Cell Development |
|  | IL17 Signaling in Psoriasis |
|  | IFN-gamma/TNF-alpha Mediated Cell Proliferation in Psoriasis |
|  | Melanocyte Role in Skin Aging |
|  | POMC Activation in Keratinocytes |
|  | Th0-Cell Aberrant Activation in Systemic Lupus Erythematosus |
|  | B-Cell Activation in Systemic Lupus Erythematosus |
|  | Oxidative Stress in Vitiligo |
|  | Vitiligo |
|  | Apoptosis of Melanocyte in Vitiligo |
|  | Toll-like Receptors in beta-Cell |
|  | Macrophage Function in Diabetes Mellitus Type 1 |
|  | beta-Cell Death in Diabetes Mellitus Type 2 |
|  | Podocyte Dysfunction in Diabetic Nephropathy |
|  | AGE Related Insulin Resistance in Skeletal Muscles |
|  | beta-Cell Destruction through Cytokines in Diabetes Mellitus |
|  | beta-Cell Recovery in Diabetes Mellitus |
|  | beta-Cell Destruction in Diabetes Mellitus Type 1 |
|  | Nitric Oxide Effects on beta-Cell |
|  | Hyperglycemia and Hyperlipidemia Trigger beta-Cell Apoptosis |
|  | beta-Cell Function Inhibition by Ciclosporine and Tacrolimus (Rodent Model) |
|  | FOXO1 and SREBP-1C Role in beta-Cell Suppression (Rodent Model) |
|  | Immune Sustem Activation in Hashimoto's Thyroiditis |
|  | Hashimoto's Thyroiditis |
|  | Apoptosis of Thyroid Cell in Hashimoto's Thyroiditis |
|  | Hashimoto's Thyroiditis Triggers |
|  | Myocyte Dysfunction in Hypoparathyroidism |
|  | Thymic Follicular Hyperplasia |
|  | Fatty Acids in Hypertension and Atherosclerosis Development |
|  | Cetuximab Resistance in Colorectal Cancer |
|  | Metastatic Colorectal Cancer |
|  | EGFR Nuclear Signaling in Colorectal Cancer |
|  | Th17-Cell Activation in Crohn's Disease |
|  | Macrophage Dysfunction |
|  | Genes with Susceptibility for Crohn's Disease and Ulcerative Colitis |
|  | Apoptosis through VacA in Helicobacter Pylori Infection |
|  | CagA Phosphorylation Dependent Signaling |
|  | Peutz-Jeghers Syndrome |
|  | Epithelial Cell in the Innate Immune Response in Ulcerative Colitis |
|  | Th2-Cell Function in Ulcerative Colitis |
|  | T-Cell Cytotoxic Response in Ulcerative Colitis |
|  | Apoptosis Block in Acute Myeloid Leukemia Cell |
|  | Acute Myeloid Leukemia |
|  | B-Cell Acute Lymphoblastic Leukemia |
|  | B-Cell Chronic Lymphocytic Leukemia |
|  | Chronic Myeloid Leukemia |
|  | Diffuse Large-B-Cell Lymphoma |
|  | Diffuse Large-B-Cell Lymphoma ABC Subtype |
|  | Lymphoma, GCB Subtype |
|  | Primary Mediastinal B-cell Lymphoma (PMBL) Subtype |
|  | Follicular Lymphoma |
|  | Hodgkin Lymphoma |
|  | Mantle Cell Lymphoma |
|  | Apoptosis of Mantle Cell Deregulation |
|  | mTOR/NF-kB/BCR Signaling Disregulation in Mantle Cell Lymphoma |
|  | Hedgehog Signaling in Mantle Cell Lymphoma |
|  | Mucosa-Associated Lymphoid Tissue (MALT) Lymphoma |
|  | Splenic Marginal Zone Lymphoma |
|  | TNFR/NF-kB Classical Pathway in Multiple Myeloma Plasma Cells |
|  | Osteoclast Activation in Multiple Myeloma |
|  | IL6/IGF1/VEGFA Signaling in Multiple Myeloma |
|  | Multiple Myeloma |
|  | TNFR/NF-kB Alternative Pathway in Multiple Myeloma Plasma Cells |
|  | T-Cell Acute Lymphoblastic Leukemia |
|  | SERPINA1 Associated Pulmonary Emphysema |
|  | SERPINA1 Associated Liver Damage |
|  | Hepatocellular Carcinoma |
|  | TP53 Signaling in Hepatocellular Carcinoma |
|  | Growth Factor Signaling in Hepatocellular Carcinoma |
|  | Pancreatic Ductal Carcinoma |
|  | Growth Factor Signaling in Pancreatic Neoplasms |
|  | TP53 Signaling in Pancreatic Neoplasms |
|  | Pancreatic Neuroendocrine Tumors |
|  | STK11 Signaling in Pancreatic Neoplasms |
|  | TGFBR Signaling in Pancreatic Neoplasms |
|  | Eosinophil Survival in Asthma |
|  | TSLP Signaling in Bronchial Epithelial Cell in Asthma |
|  | Basophil Activation in Asthma |
|  | Osteoclast Activation in Gout |
|  | Apoptosis of Chondrocyte |
|  | Osteoclast Activation in Psoriatic Arthritis |
|  | Keratinocyte Activation in Psoriatic Arthritis |
|  | Osteoclast Activation in Rheumatoid Arthritis |
|  | Apoptosis Block in Synovial Fibroblast |
|  | FAS Mediated Apoptosis in Systemic Scleroderma |
|  | Apoptosis Block in Infected Cells |
|  | CD4+ T-Cell Death in HIV |
|  | TNF Receptor Associated Periodic Syndrome |
|  | Li-Fraumeni Syndrome |
|  | Hereditary Breast and Ovarian Cancer Syndrome |
|  | Cowden Syndrome |
|  | PPARGC1A Repression in Huntington Disease |
|  | Glutamate Toxiicity Effects on Striatum Medium Spiny Neurons in Hantington's Disease |
|  | NF-kB Signaling Activation in Huntington Disease |
|  | Huntingtin Gene Mutation in Striatal Neuron |
|  | ER Stress in Huntington Disease |
|  | CA4 Induces Apoptosis of Choriocapillaris Endothelial Cells in RP |
|  | Oxytocin Signaling in Brain Nerve Cell (Hypothesis) |
|  | Drugs Targets in Autistic Disoreder (Hypothesis) |
|  | Werner Syndrome (Adult Progeria) |
|  | IL6 Promotes Inflammation in Muscular Dystrophies |
|  | Ca2+ Overload in Muscular Dystrophies |
|  | Muscular Dystrophies |
|  | Dystrophin Glycoprotein Complex Signaling in Muscular Dystrophies |
|  | APP and Glutamate Signaling Cause Neuronal Dysfunction in Alzheimer Disease |
|  | Apoptosis and Immediate Early Gene Activation in Epileptiform Disorders |
|  | Glioblastoma, Primary |
|  | Glioblastoma, Secondary |
|  | Astrocytoma |
|  | Ependymoma |
|  | Medulloblastoma |
|  | Growth Factor Signaling in Neuroblastoma |
|  | Neuroblastoma |
|  | ALK Associated Neuroblastoma |
|  | Sonic Hedgehog Signaling in Neuroblastoma |
|  | non-Hereditary Genetic Rearrangements in Neuroblastoma |
|  | SOD1 Mutation |
|  | Glutamate Mediated Excitotoxicity in Amyotrophic Lateral Sclerosis |
|  | MIR155 Role in Th17-Cell in Multiple Sclerosis |
|  | SPP1 Induced Survival of Myelin Specific T-Cells in Multiple Sclerosis |
|  | ERBB/VEGFR/Akt Signaling in Breast Cancer |
|  | IGF1R/AKT Signaling in Breast Cancer |
|  | ESR1/ERBB Positive Luminal Breast Cancer |
|  | Breast Cancer |
|  | ESR1 Signaling in Breast Cancer |
|  | Endometrial Cancer |
|  | Endometrioid Endometrial Cancer |
|  | Clear Cell Endometrial Cancer and Papillary Serous Endometrial Cancer |
|  | Dioxin Role in Endometriosis |
|  | Progesterone Resistance in Endometriosis |
|  | Retinoic Acid Role in Endometriosis |
|  | Endometrioid Ovarian Carcinoma |
|  | Clear Cell Ovarian Carcinoma |
|  | High-Grade Serous Ovarian Carcinoma |
|  | Low-Grade Serous Ovarian Carcinoma |
|  | Ovarian Cancer |
|  | Mucinous Ovarian Carcinoma |
|  | FOXL2 Mutation in Ovarian Dysgenesis |
|  | MIF Role in Cholesteatoma |
|  | IL6/JAK/STAT3 Signaling in Cholesteatoma |
|  | HMGB1/RAGE Signaling in Middle Ear Cholesteatoma |
|  | Subepithelial Fibroblasts in Middle Ear Cholesteatoma |
|  | Cholesteatoma |
|  | HPV E1 and E2 Expression in Early Phase of Cancer |
|  | HPV E6 and E7 Induced Block of Apoptosis in Keratinocytes |
|  | HPV Infection and Cancer |
|  | HPV E6 and E7 Induced Disruption of TNFR/NF-kB Signaling in Keratinocytes |
|  | HPV E5 Regulates EDNRA and EGFR Signaling Pathway in Keratinocytes |
|  | Androgen Receptor/Akt Signaling |
|  | Fatty Acid Synthase (FASN) Signaling |
|  | Androgen Receptor/FKBP5 Signaling |
|  | Androgen Receptor/NKX3-1 Signaling |
|  | Kruppel-like Factor 6 Signaling in Prostate Cancer |
|  | Prostate Cancer |
|  | RNase L Signaling in Prostate Cancer |
|  | Androgen Receptor/Protein Kinase C-delta Signaling |
|  | Androgen Receptor/SGK1 Signaling |
|  | Oxidative Stress, All-Trans-Retinal and Lipofuscin Toxicity in AMD |
|  | TLR3 Role in Retinal Pigment Epithelial Cell |
|  | ER Stress in Macular Degeneration |
|  | VEGFA in Neovascular Glaucoma |
|  | OPTN Effects on NF-kB Signaling in Glaucoma |
|  | MYOC Associated Glaucoma |
|  | Glutamate Mediated Excitotoxicity in Glaucoma |
|  | OPTN Associated Glaucoma |
|  | Neurotrophic Factor Deprivation in Retinal Ganglion Cell Death |
|  | Aminoglycosides and Cisplatin Induced Ototoxicity (Mouse Model) |
|  | Alveolar Epithelial Cell Apoptosis in COPD |
|  | Alveolar Epithelial Cell Autophagy in COPD |
|  | Podocyte Dysfunction in Glomerulonephritis |
|  | Apoptosis of Renal Tubule Epithelial Cell in Pyelonephritis |
|  | GLP1R -> ARRB1 Signaling |
|  | AGTR1 -> ARRB1/ARRB2 Signaling |
|  | Tamoxifen Induced Endometrial Cancer |
|  | Dexamethasone Induced Osteoporosis |
|  | Doxorubicin Induced Cardiotoxicity |
|  | TAM Receptor Signaling Activation in Cancer |
|  | PI3K/AKT/MTOR Signaling Activation in Cancer |
|  | DREAM Complex and FOXM1/MYBL2 Promote Cell Cycle Progression in Cancer |
|  | Hedgehog Signaling Activation in Cancer |
|  | NFKB Canonical Signaling Activation in Cancer |
|  | RAS/RAF/MAPK Signaling Activation in Cancer |
|  | NFKB Non-Canonical Signaling Activation in Cancer |
|  | DNA Damage Checkpoint Impairment in Cancer |
|  | STAT3 and NFkB Activate Inflammation-Induced Tumorigenesis |
|  | RAS/RAF/MAPK Signaling Activation by Blocking of Tumor Suppressors |
|  | Hedgehog Signaling Activation by Blocking of Tumor Suppressors |
|  | PI3K/AKT/MTOR Signaling Activation by Blocking of Tumor Suppressors |
|  | NFKB Signaling Activation by Blocking of Tumor Suppressors |
|  | Apoptosis Block by Inhibitor of Apoptosis Proteins (IAPs) in Cancer |
|  | Suppression of Caspase Activity in Cancer |
|  | Apoptosis Block by Death Receptor Signaling in Cancer |
|  | Apoptosis Evasion in Cancer: Overview |
|  | Hypoxia-Induced Cell Death Evasion in Cancer |
|  | Apoptosis Block by BCL2 Family Proteins in Cancer |
|  | TERT Activation in Cancer |
|  | Telomeric Proteins/NFKB Positive Feedback Interplay in Cancer |
|  | Oxidized LDL/OLR1 Activates Cell Proliferation in Cancer |
|  | ANGPT/TEK Maintains Endothelial Cell Quiescence in Cancer |
|  | Integrins in Cancer Cell Motility, Invasion and Survival |
|  | BSG (CD147) in Cancer Cells Motility, Invasion and Survival |
|  | Hyaluronic Acid, CD44 and HMMR in Cancer Cell Invasion and Survival |
|  | Respiratory Chain Impairment in Cancer |
|  | Skeletal Muscle Wasting in Cancer Cachexia |
|  | Glutamine in Cancer Metabolism |
|  | Mutations in Krebs Cycle Enzymes in Cancer |
|  | Effector T-cell Inactivation in Cancer Immune Escape |
|  | TNF-alpha/TNFRSF1A Signaling |
|  | FOXO1 Signaling |
|  | Ras Signaling |
|  | AXL Receptor Tyrosine Kinase Signaling |
|  | mTOR Signaling |
|  | KRAS Signaling |
|  | VEGF Signaling |
|  | EGFR Signaling |
|  | HRAS Signaling |
|  | NF-kB Canonical Signaling |
|  | ROS in Neutrophil Mediated Cell Damage |
|  | ER Stress (Unfolded Protein Response) |
|  | Glutamate Mediated Neurotoxicity |
|  | Mitochondrial Damage Induced by Hypoxia |
|  | Ca2+ Cytosolic Overload |
|  | ace2 |
|  | New Pathway (2) |
|  | sialic |
|  | Histones Modifications Sites in Cell Vitality |
|  | H3 Arginin Modification Sites |
|  | H2A Modification Sites |
|  | H4 Modification Sites |
|  | Overview. Diseases of the digestive system (chapter 10) |
|  | Overview. Diseases of the eye (chapter 6) |
|  | 2\_Interleukin-17 and interleukin-22 signaling in psoriasis |
|  | 3\_X-linked ichthyosis overview |
|  | 2\_Chondrocyte death in osteoarthritis |
|  | 2\_Podocyte dysfunction in glomerulonephritis |
|  | 2\_Progesterone resistance stimulates endometriotic cell survival |
|  | 3\_Role of neurotrophic factor deprivation in retinal ganglion cell death |
|  | 2\_MYOC-associated glaucoma |
|  | 2\_Oxidative stress, all-trans-retinal, and lipofuscin toxicity in agerelated macular degeneration |
|  | 3\_Endoplasmic reticulum stress in age-related macular degeneration |
|  | 1\_Insufficient activation of immune response in the middle ear epithelium cells in otitis media |
|  | 1\_Low-density lipoprotein internalization and the accumulation of lipids in atherosclerosis |
|  | 1\_Endothelial cells stimulate vasoconstriction and thrombosis in pulmonary hypertension |
|  | 2\_Cigarette smoke and inflammation cause alveolar epithelial cell death in COPD |
|  | 2\_2\_Eosinophilia and neutrophilia in asthma: eosinophil apoptosis |
|  | 4\_2\_Airway smooth muscle cells proliferation and airway tissue remodeling |
|  | 3\_Autophagy dysfunction in Crohn's disease |
|  | 1\_Intestine epithelial cell dysfunction in ulcerative colitis |
|  | 2\_Lipotoxicity in NAFLD |
|  | 6\_p12I Viral protein promotes T-cell activation in HTLV-1 infection |
|  | 2\_Human papilloma virus E1 and E2 proteins in virus replication |
|  | 3\_Human papilloma virus E4 and E5 proteins in viral reproduction |
|  | 4\_Human papilloma virus E6 and E7 proteins promote epithelial cell survival |
|  | 5\_Human papilloma virus E6 and E7 proteins promote epithelial cell proliferation |
|  | 2\_HIV - induced depletion of CD4+ T-cells |
|  | 3\_2\_HIV postintegration latency |
|  | 2\_Hyperglycemia and hyperlipidemia trigger beta-cell apoptosis |
|  | 1\_SERPINA1-associated pulmonary emphysema |
|  | 2\_SERPINA1-associated liver damage |
|  | 3\_Islet beta-cell destruction in T1D |
|  | 2\_Neuron and Schwann cell death |
|  | 1\_Glutamate and calcium excitotoxicity of motor neurons in ALS |
|  | 2\_Mutations in SOD1 and associated proteins cause motor neuron death in familial ALS |
|  | DIPG non histone mutations |
|  | New Pathway (1) |
|  | Interleukin-17 and interleukin-22 signaling in psoriasis |

---

|  |  |
| --- | --- |
| MedScan ID | 4000007 |
|  | 4112501 |
|  | 4106917 |

---

|  |  |
| --- | --- |
| Alias | programmed cell death |
|  | commitment to apoptosis |
|  | apoptotic cellular deaths |
|  | apoptotic death |
|  | apoptosis signalings |
|  | apoptotic-like cell deaths |
|  | apoptotic death of the cell |
|  | cell suicide |
|  | apoptotic programs |
|  | apoptotic cell death |
|  | apo-ptosis |
|  | intrinsic mitochondrial pathways |
|  | signaling of apoptosis |
|  | apoptotic deaths |
|  | intrinsic mitochondrial pathway |
|  | apoptotic suicides |
|  | mitochondrial pathways |
|  | cell suicides |
|  | apoptotic cell deaths |
|  | apoptotic pathway |
|  | cellular suicides |
|  | suicidal cell death |
|  | apoptoses |
|  | apoptotic alteration |
|  | apoptotic pathways |
|  | apoptotic signalings |
|  | ap-optosis |
|  | cellular suicide |
|  | apoptosis |
|  | mitochondria-mediated cell death |
|  | apoptosis signaling |
|  | apoptotic program |
|  | apoptotic-like cell death |
|  | signaling during apoptosis |
|  | apoptotic cellular death |
|  | apoptotic alterations |
|  | programmed cell deaths |
|  | apoptotic signaling |
|  | apoptotic suicide |
|  | apoptotic |
|  | suicidal cell deaths |
|  | mitochondrial pathway |
|  | mPT-mediated cell death |
|  | non apoptotic programmed cell death |
|  | programmed cell death type I |
|  | nonapoptotic programmed cell death |

---

|  |  |
| --- | --- |
| GO ID | 0006915 |

---

|  |  |
| --- | --- |
| LocatorString | pmquery:prevacid, prilosec (omeprazole) 2002:2010[PDAT] |
|  | pmftq:oncomed |
|  | pmquery:curcumin+prostate+cancer |
|  | pmquery:mir122+OR+mir-122 |
|  | pmquery:curcumin prostate cancer |

---
